# GourmetNet: Food Segmentation Using Multi-Scale Waterfall Features with Spatial and Channel Attention

**DOI:** 10.3390/s21227504

**Published:** 2021-11-11

**Authors:** Udit Sharma, Bruno Artacho, Andreas Savakis

**Affiliations:** Department of Computer Engineering, Rochester Institute of Technology, Rochester, NY 14623, USA; us2848@g.rit.edu (U.S.); bmartacho@mail.rit.edu (B.A.)

**Keywords:** semantic segmentation, food segmentation, multi-scale features, spatial attention, channel attention

## Abstract

We propose GourmetNet, a single-pass, end-to-end trainable network for food segmentation that achieves state-of-the-art performance. Food segmentation is an important problem as the first step for nutrition monitoring, food volume and calorie estimation. Our novel architecture incorporates both channel attention and spatial attention information in an expanded multi-scale feature representation using our advanced Waterfall Atrous Spatial Pooling module. GourmetNet refines the feature extraction process by merging features from multiple levels of the backbone through the two attention modules. The refined features are processed with the advanced multi-scale waterfall module that combines the benefits of cascade filtering and pyramid representations without requiring a separate decoder or post-processing. Our experiments on two food datasets show that GourmetNet significantly outperforms existing current state-of-the-art methods.

## 1. Introduction

Semantic segmentation is an important computer vision task that has advanced significantly due to deep learning techniques [1,2,3,4,5,6]. Most semantic segmentation methods focus on standard datasets, such as MS-COCO [7] and Cityscapes [8], but there is great potential in diverse applications such as remote sensing [9], agriculture [10] and food recognition [11,12]. Unfortunately, methods for food segmentation are still lagging in development and this paper aims to advance the state-of-the-art.

Food segmentation methods are useful in a variety of applications including nutrition monitoring [13,14,15], food volume estimation [16,17], calorie estimation [18,19], ingredient detection [20,21], recipe generation [22,23] and food preparation. The application of nutrition monitoring using smartphones can significantly benefit from accurate food segmentation by alleviating the user from manually entering food labels and portion size for each meal. In this context, the user takes a picture of the meal and food segmentation automatically detects each food item and provides an estimate of the portion size. This information can be further used to assess the nutritional content of a meal and monitor the nutrition intake of an individual over a time period in order to provide recommendations for dietary improvements for health benefits. This scenario is supportive of the World Health Organization’s Sustainable Development Goals (SDGs) to achieve improved nutrition, ensure sustainable consumption patterns, ensure healthy lives and promote well-being for all at all ages.

Food segmentation is a challenging problem due to high intra-class variability, that is, a food element can be presented in a widely diverse set of shapes, sizes, colors, and combinations with other ingredients. Another characteristic of food analysis is that some food items are routinely paired, allowing the network to infer correlations between the occurrence of different classes.

Early food segmentation works were based on traditional computer vision methods [24,25]. Segmentation of food images was performed in a deep learning framework as an initial step towards calorie estimation in im2calories [19]. However, the dataset in im2calories was not made public for further research. The UNIMIB2016 dataset [26] was introduced for food segmentation with polygon annotations for 73 food categories. Initial segmentation results were obtained in [11,12] based on the popular SegNet [4] and DeepLab [5] methods respectively. Another publicly available dataset is the UEC FoodPix dataset [27], where DeepLabv3 [28] was used to perform semantic segmentation. Our approach employs attention mechanisms on multi-scale waterfall features and significantly outperforms the current state-of-the-art in the aforementioned datasets.

**Figure 1:**
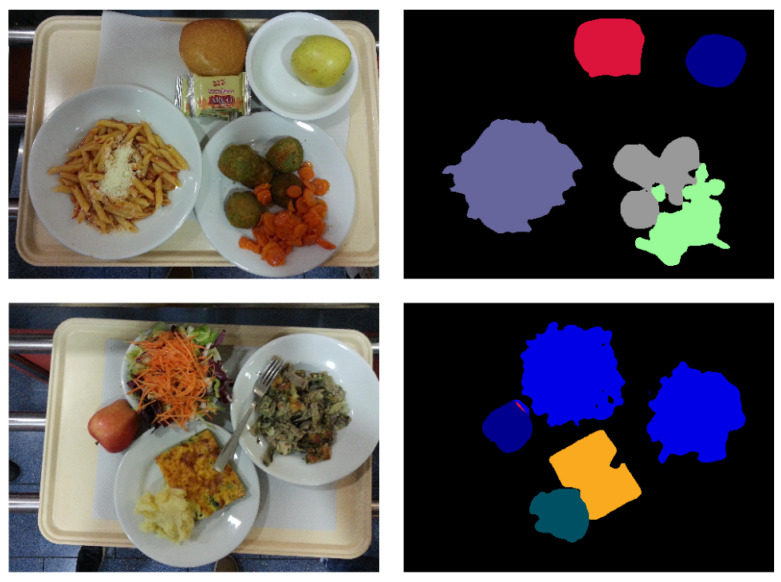
Food segmentation examples using GourmetNet.

We propose GourmetNet, a single-stage network for food segmentation, that is end-to-end trainable and generates state-of-the-art results without requiring multiple iterations, intermediate supervision or postprocessing. Our method is inspired by recent advances in multi-scale feature representations [6,29] and dual attention methods [30] to create a contextual multi-scale framework that improves the pixel-level detection of different foods for segmentation. Examples of food segmentation obtained with GourmetNet are shown in Figure 1.

The main aspect of our novel architecture is the extraction of both channel and spatial attention information for an expanded multi-scale feature representation using the advanced Waterfall Atrous Spatial Pooling (WASPv2) module [29]. The WASPv2 module generates multi-scale features by increasing the Field-of-View (FOV) for the network while better describing shapes, colors and textures from images, resulting in a significant improvement in accuracy for food segmentation.

GourmetNet predicts the location of multiple food classes and performs segmentation of multiple food items based on contextual information due to the multi-scale feature representation. The contextual approach allows our network to include information from the entire image, including all channels and shapes, and consequently does not require post analysis based on statistical or geometric methods, for example, there is no need to use the computationally expensive Conditional Random Fields (CRF).

The main contributions of this paper are the following:

- We propose GourmetNet, a single-pass, end-to-end trainable, multi-scale framework with channel and attention modules for feature refinement;
- The integration of channel and attention modules with waterfall spatial pyramids increases performance due to improved feature extraction combined with the multi-scale waterfall approach that allows a larger FOV without requiring a separate decoder or post-processing.
- GourmetNet achieves state-of-the-art performance on the UNIMIB2016 and UEC FoodPix food segmentation datasets. The GourmetNet code is shared on github (https://github.com/uditsharma29/GourmetNet (accessed on 8 November 2021)).

The rest of this paper is organized as follows. After the introduction, related work on food segmentation, multi-scale features and attention mechanisms is overviewed in Section 2. The proposed GourmetNet framework and its components, including the channel attention module, the spatial attention module, and the waterfall module, is presented in Section 3. Experimental methods, datasets and evaluation metrics are discussed in Section 4. Results of ablation studies, comparisons with the state-of-the-art, and representative examples are shown in Section 5. Conclusions and future work are outlined in Section 6.

## 2. Related Work

Semantic segmentation methods have improved significantly following the breakthrough introduction of the Deconvolution Network [2] and Fully Convolutional Networks (FCN) [1]. The U-Net architecture [3] extended the convolution-deconvolution framework by concatenating features from the convolution layers with their counterparts in the deconvolution part of the network. Using an encoder–decoder approach, SegNet [4] used the initial layers of the VGG backbone [31] in the encoder stage with up-sampling deconvolution layers in the decoder stage. SegNet was further developed in [32] to include Bayesian techniques to model uncertainty. Aiming to expand the learning context of the network, Pyramid Scene Parsing (PSPnet) [33] combined scene parsing with semantic segmentation. The Efficient Network (ENet) approach [34] sought to develop a real-time semantic segmentation method, resulting in a significant improvement in processing speed compared to other methods.

DeepLab [5] is a popular architecture that proposed the Atrous Spatial Pyramid Pooling (ASPP) module, leveraging the use of atrous (dilated) convolutions [35] and Spatial Pyramid Pooling (SPP) [36]. ASPP incorporates branches with different rates of dilation for their convolutions, increasing its field of view and better learning global context. DeepLabv3 [28] improved this approach by applying atrous convolutions in a cascade manner, progressively increasing the dilation rates through the layers. A further improvement was reported in the DeepLabv3+ [37] which adds a simple but effective decoder to the architecture in DeepLabv3 and uses separable convolutions to decrease the computational cost of the network without a significant drop in performance.

### 2.1. Waterfall Multi-Scale Features

The Waterfall Atrous Spatial Pooling (WASP) module was introduced in WASPnet [6] for semantic segmentation. The WASP module was designed to leverage the reduced size of cascaded atrous convolutions while maintaining the larger FOV through multi-scale features in the pyramid configuration. The WASP architecture effectively addressed the issue of high memory requirement present on the ASPP module, and reduced parameters by over 20% while improving improve segmentation performance compared to the original ASPP architecture used in DeepLab. Additionally, the WASP multi-scale feature extraction was found to be useful for human pose estimation and generated state-of-the-art results with the UniPose method [38].

An improved version of the WASP module, named WASPv2, was proposed for the task of multi-person pose estimation in the OmniPose framework [29]. This new feature extraction model combines the learning of the multi-scale features using the waterfall approach while making use of low-level features from the backbone to embed spatial information and maintain high resolution throughout its layers. The WASPv2 module shows increased performance for pose estimation and further reduction in computational cost, presenting promising potential to be applied for semantic segmentation. In this paper, we adopt the WASPv2 module and re-purpose it with channel and spatial attention for semantic segmentation in GourmetNet.

### 2.2. Attention Mechanisms

Attention was initially proposed in sequence-to-sequence (seq2seq) models for neural machine translation [39,40]. The introduction of the transformer model [41] is a significant breakthrough in Natural Language Processing (NLP), where the multi-head self-attention layer in the transformer aligns words to obtain a representation of the sequence. The attention approach was expanded to computer vision tasks in [42], by using a Recurrent Neural Network (RNN) to associate generated words with certain parts of the image.

The use of attention to improve semantic segmentation methods was explored by [43], taking the approach of training attention heads across scales for semantic segmentation. Similarly, the Dual Attention Network (DANet) [44] uses the channel and spatial attention to improve the network’s understanding of the global context for the image. The method in [45] performs the reverse operation for attention, also aiming to better understand the entire context of the image.

Expanding on attention decoders, BiSeNet [46] fuses two branches for low and high level features bilaterally aiming to construct a real-time approach for segmentation. In similar fashion, the Dual Attention Decoder [30] applies the low-level features to perform its attention module on high level features while creating a channel mask to its low-level features. GourmetNet leverages the promising use of attention to further improve its multi-scale approach.

### 2.3. Food Segmentation

Food segmentation methods were initially developed using traditional computer vision techniques. Local variation and normalized graph cut [47] were used by [24] to extract the segmentation. The approach in [25] focused on the color and shape of the food items based on the JSEG segmentation [48], which contains two independent steps: color quantization and spatial segmentation. The biggest challenges for food segmentation and related tasks, such as volume estimation, are due to its high intra-class variability regarding texture, density, colors, and shapes.

Deep learning based methods have proven to be more effective than rule based techniques for food segmentation. Initial applications for food segmentation with deep learning include the mobile application of im2calories [19], having a long list of non-integrated steps for the food segmentation task. This method relies on the GoogleNet model [49] to detect instances of food, followed by another GoogleNet model trained to detect the food type, and finally performs pixel level semantic classification with DeepLab [5].

In addition to introducing the UEC Foodpix dataset, [27] proposes a multi-step approach for food segmentation by applying YOLOv2 [50] for food detection followed by segmentation using the DeepLabv3 method [28] with an Xception net backbone [51].

Slightly increasing the integration of networks for the task of food segmentation, Reference [52] applies an encoder–decoder architecture to perform binary segmentation on food images. The method combines the first three layers of the ResNet-101 [53] and a decoder. SegNet [4] and DeepLab [5] architectures are adopted by [11,12] respectively to perform semantic segmentation on the UNIMIB2016 dataset [26].

## 3. Proposed Method

**Figure 2:**
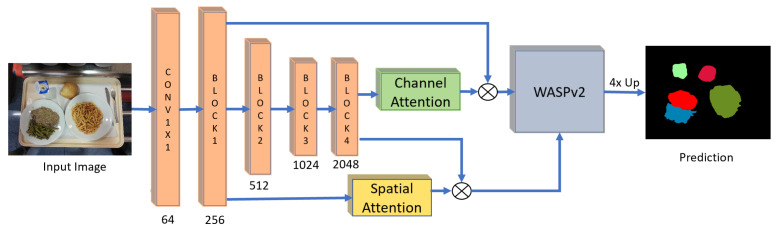
The proposed GourmetNet architecture for food segmentation. The input image is fed through a modified ResNet backbone and the features are refined by the spatial and channel attention modules before the multi-scale WASPv2 module which produces the output semantic segmentation result. The numbers below each block indicate the number of feature channels.

The proposed GourmetNet framework, illustrated in Figure 2, is a single pass, end-to-end trainable network for food segmentation. Inspired by [30], we introduce attention mechanisms with the multi-scale feature extraction of the WASPv2 module. GourmetNet re-purposes the use of the dual attention module to extract context prior to the multi-scale feature extraction and decoder stage from the WASPv2 module and the spatial pooling modules.

We determine that attention is more useful when it operates on features coming directly from the backbone, as opposed to waiting until after the feature extraction during the spatial pooling modules. This is done because features from the backbone are richer in information and the attention modules have more to work with. Further, GourmetNet combines the improvements in feature representations from WASPv2 and the attention extraction of information from both channel and spatial attention modules.

The processing pipeline of GourmetNet is shown in Figure 2. The low-level features are extracted from the input image through the first block of a modified ResNet feature extractor and include a dilated last block for the generation of a large FOV. The high-level features are the output of the last block of the modified ResNet feature extractor. All features are then processed through the attention modules in order to better extract the spatial understanding from the low-level features and richer contextual information from the high-level features.

### 3.1. Backbone

We employ the ResNet backbone modified with atrous convolutions as done in [5]. For feature extraction, the first four blocks of ResNet-101 are used. However, the last block is modified for multi-scale feature learning. Instead of using regular convolutions, this block uses atrous convolutions. Further, each convolution in this block uses different rates of dilation to capture multi-scale context. The output size of the feature maps is determined by the output stride. For an output stride of s, the output is reduced by s times from the original image. Having a higher output stride affects the quality of dense predictions but reduces the size of the model. For practical reasons, we use an output stride of 16 in our experiments.

### 3.2. Attention Modules

GourmetNet utilizes two attention modules to generate masks and refine the low-level and high-level features extracted from the modified ResNet backbone. The placement of the attention modules in the GourmetNet framework is illustrated in Figure 2. The spatial attention branch uses the low-level features from the backbone to create a mask containing spatial information to refine the high-level features prior to the waterfall module. The channel attention branch uses the high-level features to create a mask containing channel information from the feature maps, and applies it to refine the the low-level features.

The dimensions of the generated spatial mask are h×w×1, where *h* and *w* are the height and width of the low-level feature maps. The same mask is broadcast across all feature maps in the high-level features space.

#### 3.2.1. Channel Attention

Channel attention utilizes high-level features which consist of 2048 feature maps with width and height reduced by a factor of four compared to the original dimensions of the input image. Our modified channel attention module progressively reduces the number of feature maps to 256. These maps produce the channel attention mask used as one of the inputs to the WASPv2 module after pixel-wise multiplication with the low-level features from the backbone.

**Figure 3:**
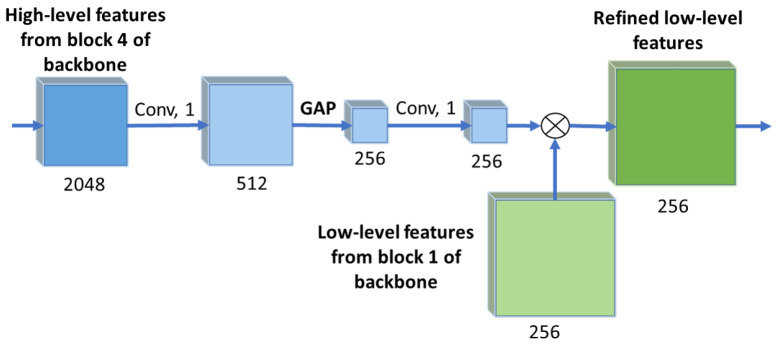
Channel attention module architecture. The high-level features from the backbone are fed to a 1 × 1 convolution to reduce the number of maps to 512, followed by a global average pooling layer (GAP) and another 1 × 1 convolution, generating 256 maps. These maps are then multiplied with the low-level features from the backbone, generating the refined low-level features. The numbers below each block indicate the number of feature channels.

The channel attention module architecture is shown in Figure 3. The 2048 high-level feature maps from the modified ResNet backbone are processed with 1 × 1 convolutions to reduce the number of feature maps to 512, followed by a global average pooling layer and another 1 × 1 convolution stage, reducing the number of feature maps to 256. The output of the module is then multiplied pixel-wise with the low-level features from the backbone, producing the refined low-level features with 256 channels. The channel attention module operation can be expressed as follows:

(1) $$f_{rl}=f_{l}*(K_{1}⊛AP\left(K_{1}⊛f_{h}\right),$$

where ⊛ represents convolution, $f_{rl}$ represents the refined low-level features, $f_{l}$ are the low-level features extracted from block 1 of the backbone, ∗ represents element-wise multiplication, $K_{1}$ is a kernel of size 1 × 1, *AP* denotes Average Pooling, and $f_{h}$ represents the high-level features extracted from backbone. The dimensions of the channel mask are 1×1×c where *c* is the number of channels in the low-level feature space. This mask is broadcast to all the pixels in the low-level feature maps.

#### 3.2.2. Spatial Attention

Spatial attention utilizes low-level features that are extracted from the first block of the modified ResNet backbone, by converting features maps into the spatial attention mask. This mask is then used to refine the high-level backbone features using element-wise multiplication.

**Figure 4:**
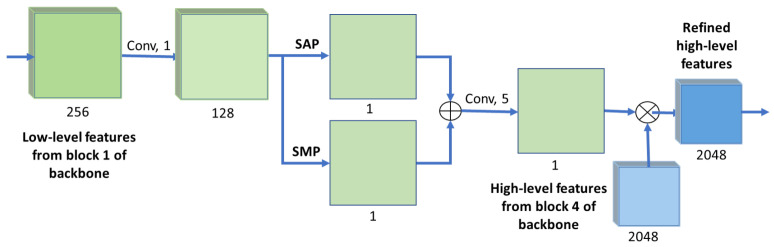
Spatial attention module architecture. The low-level features from the backbone are fed to a 1 × 1 convolution to reduce it to 128 maps. The maps are then fed to both a SAP and SMP layers, with their respective results being added. A final 5 × 5 convolution is used prior to the multiplication with the high-level features from the backbone, resulting in the refined high-level features for GourmetNet. The numbers below each block indicate the number of feature channels.

The spatial attention module is shown in Figure 4. It receives the 256 channels of low-level features from the first block of the modified ResNet backbone, and reduces them to 128 channels via 1 × 1 convolution. This is followed by a set of two parallel pooling operations, one for spatial average pooling (SAP) and one for spatial max pooling (SMP). The outputs of both spatial pooling operations are then concatenated and processed through a 5 × 5 convolution in order to extract spatial information with a larger FOV. The output of the module is then multiplied pixel-wise with the high-level features from the backbone, producing the refined high-level features with 2048 channels. The mathematical representation of the spatial attention module can be described as follows:

(2) $$f_{rh}=f_{h}*\left(K_{5}⊛\left(SAP\left(K_{1}⊛f_{l}\right)⊕SMP\left(K_{1}⊛f_{l}\right)\right)\right),$$

where ⊛ represents convolution, $f_{rh}$ represents the refined high-level features, $f_{h}$ are the high-level features extracted from the backbone, ∗ represents element-wise multiplication, $K_{1}$ and $K_{5}$ are kernels of size 1 × 1 and 5 × 5 respectively, *SAP* and *SMP* denote Spatial Average Pooling and Spatial Max pooling operations, respectively, ⊕ is a concatenation operation, and $f_{l}$ represents the low-level features extracted from block 1 of the backbone.

### 3.3. Multi-Scale Waterfall Features

**Figure 5:**
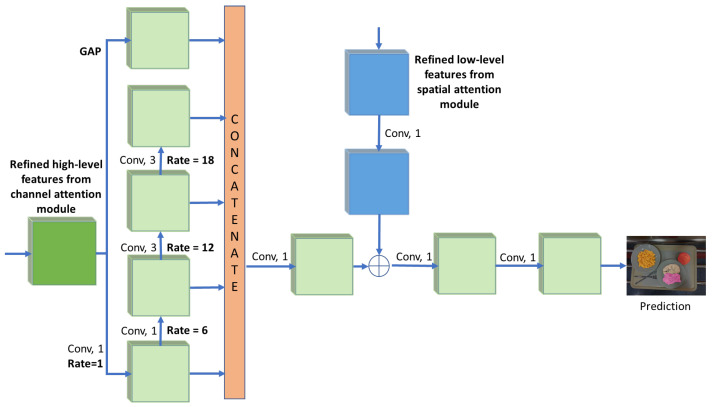
The advanced waterfall (WASPv2) module architecture with channel attention and spatial attention refined features.

Following the refinement of the low-level and high-level features via the attention modules, we perform multi-scale feature extraction and decoding through the WASPv2 module [29]. The WASPv2, depicted in Figure 5, increases the FOV by applying a set of atrous convolutions with dilation rates of [1,6,12,18] assembled in a waterfall configuration.

The waterfall architecture utilizes progressive filtering in an efficient cascade architecture, while maintaining the multi-scale FOV found in the spatial pyramid configurations. The refined low-level features are concatenated with the high-level features to obtain a multi-scale representation with increased FOV. The final layers with 1 × 1 convolutions acts as an inbuilt decoder, generating the final segmentation maps for our GourmetNet model without requiring a separate decoder module or postprocessing.

## 4. Experimental Methods

### 4.1. Datasets

We perform food segmentation experiments with GourmetNet on two datasets: the UECFoodPix dataset [27] and the UNIMIB2016 dataset [26]. The UEC FoodPix dataset is a large scale dataset for food segmentation collected by researchers in Japan. It consists of 9000 images for training and 1000 images for testing, labelled with manually annotated masks to segment 102 food categories. The main challenges of the UEC FoodPix dataset include the presence of multiple food classes on the same plate without a significant separation, diverse camera angles, various arrangements of the plates, and variation of the image size. Annotations for the UEC FoodPix dataset were generated using a coarse automated tool and manually refined by the authors [54].

The UNIMIB2016 dataset is a popular food dataset, especially for the tasks of food classification and recognition. The dataset was collected by researchers from the University of Milan, Italy, and consists of 1010 tray images that include 73 different food categories with a total of 3616 food instances. This dataset provides food region information as polygons that can be converted to masks for performing semantic segmentation. Most images contain several plates on a tray with each plate containing one food item. All images are shot from a constant angle and at the same high resolution (3264 × 2448). The dataset is divided into 650 images for training and 360 images for testing. Annotations were created using an automated tool [55] to generate polygons using the Douglas-Peucker algorithm [56]. A drawback of this annotation method is the more coarse borders resulting from the polygon method.

### 4.2. Parameter Setting

We trained GourmetNet in all experiments for 100 epochs by applying a batch size of 8. We implemented a multi-step learning rate routine with a base learning rate of $10^{-5}$ and steps of 0.3 at epochs 40 and 70. The model was trained with the Cross-Entropy (CE) loss using the Stochastic Gradient Descent (SGD) optimizer [57]. The weight decay was set to 5 ×$10^{-4}$ and momentum to 0.9 [58]. All experiments were performed using PyTorch on Ubuntu 16.04. The workstation had an Intel i5-2650 2.20 GHz CPU with 16 GB of RAM and an NVIDIA Tesla V100 GPU.

The experiments were performed with an input size of 320 × 320 for the UEC FoodPix [27] dataset and on an image size of 480 × 360 for the UNIMIB2016 [26] dataset, in order to match resolution with prior literature during accuracy comparisons. Since the code for the dual attention decoder is not publicly available, we wrote our own code based on the architecture described in [30].

### 4.3. Evaluation Metrics

The evaluation of the GourmetNet experiments was based on the Mean Intersection over Union (mIOU), a standard metric used for semantic segmentation. The *IOU* was calculated as:

(3) $$IOU=\frac{TP}{TP+FP+FN}$$

where *TP*, *FP* and *FN* represent True Positives, False Positives and False Negatives, respectively. The mIOU was obtained by the simple average score of IoU for all classes and instances in the dataset.

## 5. Results

We evaluated GourmetNet on the UEC FoodPix and UNIMIB2016 datasets, and compared our results with other methods and the previous state-of-the-art.

### 5.1. Ablation Studies

During our experiments, we performed a series of ablation studies to analyze the performance gains due to different components of GourmetNet. Table 1 and Table 2 present our ablation results on the UNIMIB2016 and the UEC FoodPix datasets. In these ablation studies GourmetNet was used with the following options: no module, Dual Attention Decoder [30], ASPP [5], WASP [6], WASPv2 [29], and our Channel Attention and Spatial Attention modules. All of the experiments were performed with a modified ResNet-101 backbone for feature extraction.

The results of Table 1 show that the mIOU performance of GourmetNet progressively increases with the inclusion of the multi-scale modules and attention modules. The WASPv2 presented the largest gain to the network as a single contribution, increasing the mIOU by 1.6% (from 68.25% to 69.17%). The dual attention decoder results in a 0.8% mIOU increase when added to the network in combination to the WASPv2 module to 70.29%. When individually utilizing our modified channel attention and spatial attention modules in addition to the WASPv2 module, the mIOU increased to 70.28% and 70.58%, respectively. The most effective configuration was found to be the inclusion of both our modified channel and spatial attention modules in addition to the WASPv2 module, resulting in the highest mIOU of 71.79% for the UNIMIB2016 dataset, a significant increase of 2.06% compared to the results obtained with Dual Attention and ASSP.

Table 2 shows the performance of GourmetNet for the UEC FoodPix dataset with the same variations in its components. Consistent with the results for the previous dataset, GourmetNet shows a progressive increase in performance with the addition of each component. The best results achieve an mIOU of 65.13% when incorporating both Channel and Spatial attention modules in addition to the WASPv2 module. The results in Table 1 and Table 2, show that the mIoU performance of GourmetNet is better for the UNIMIB2016 dataset compared to the UEC FoodPix dataset. This is due to differences between the two datasets that make UEC FoodPix more challenging, as it contains a larger number of classes, more complex boundaries between food items on the same plate and higher variation in background setting, camera angles and lighting conditions.

For completeness, we perform the experiment where we combine both the Dual Attention Decoder [30] and the channel and spatial attention modules in our proposed configuration. This configuration was not optimal, as we observe that the performance diminishes by 1.8% from 65.13% by our proposed architecture to 63.92% for the UEC FoodPix dataset (Table 2). In this configuration, we apply attention twice: once before the waterfall module and once in the dual attention decoder. However, the WASPv2 module performs better without the dual attention decoder, as indicated in the results of Table 2. A similar observation was made from the results of the UNIMIB2016 dataset in Table 1.

To assess the GourmetNet model complexity, we present the GFLOPS and the number of parameters for each configuration. These results show that the top performing WASPv2 module requires fewer parameters and is more computationally efficient than the popular ASPP architecture. The addition of the channel and spatial attention modules slightly increases the number of parameters but significantly increases the computational load.

### 5.2. Comparison to State-of-the-Art

Following our ablation studies, we compared our GourmetNet method with the current state-of-the-art for food segmentation, when results were available. We also included results using top performing methods for semantic segmentation, such as DeepLabv3+ and WASPnet. The IOU results obtained for the UNIMIB2016 dataset are shown in Table 3. GourmetNet achieves top performance, showing significant mIOU gains in comparison to other methods. For the UNIMIB2016 dataset, GourmetNet achieves 71.79% mIOU, compared to 68.87% achieved by DeepLabv3+, which is a 4.2% improvement.

**Figure 6:**
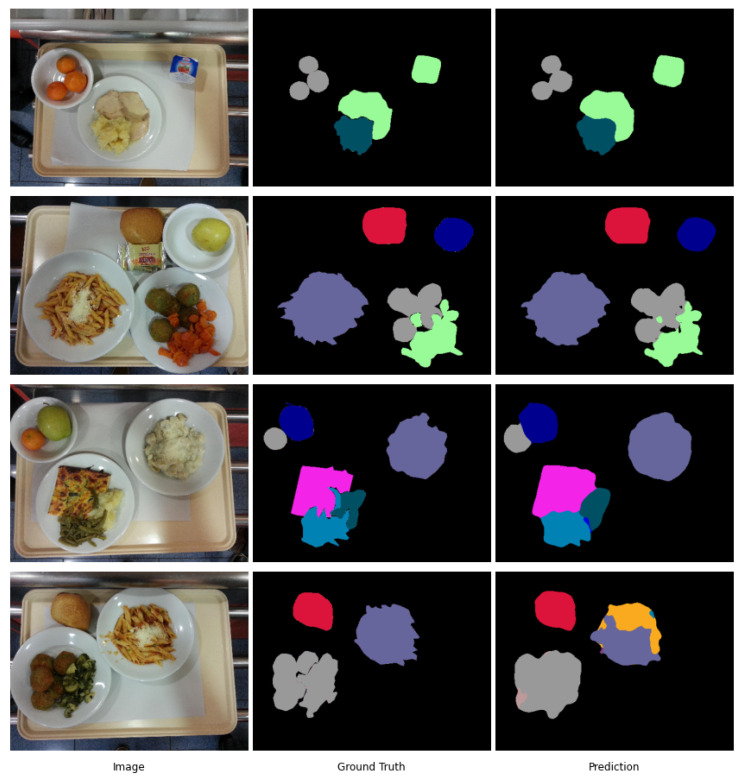
Segmentation examples using GourmetNet for the UNIMIB2016 dataset.

Example results for the UNIMIB2016 dataset are shown in Figure 6. These examples illustrate that GourmetNet successfully identifies the location of food groups with accuracy for challenging scenarios including food items that share irregular borders and shapes. Challenging conditions include the detection of food items that overlap but are described by a single segmentation mask, for example, pasta containing grated cheese on it.

**Figure 7:**
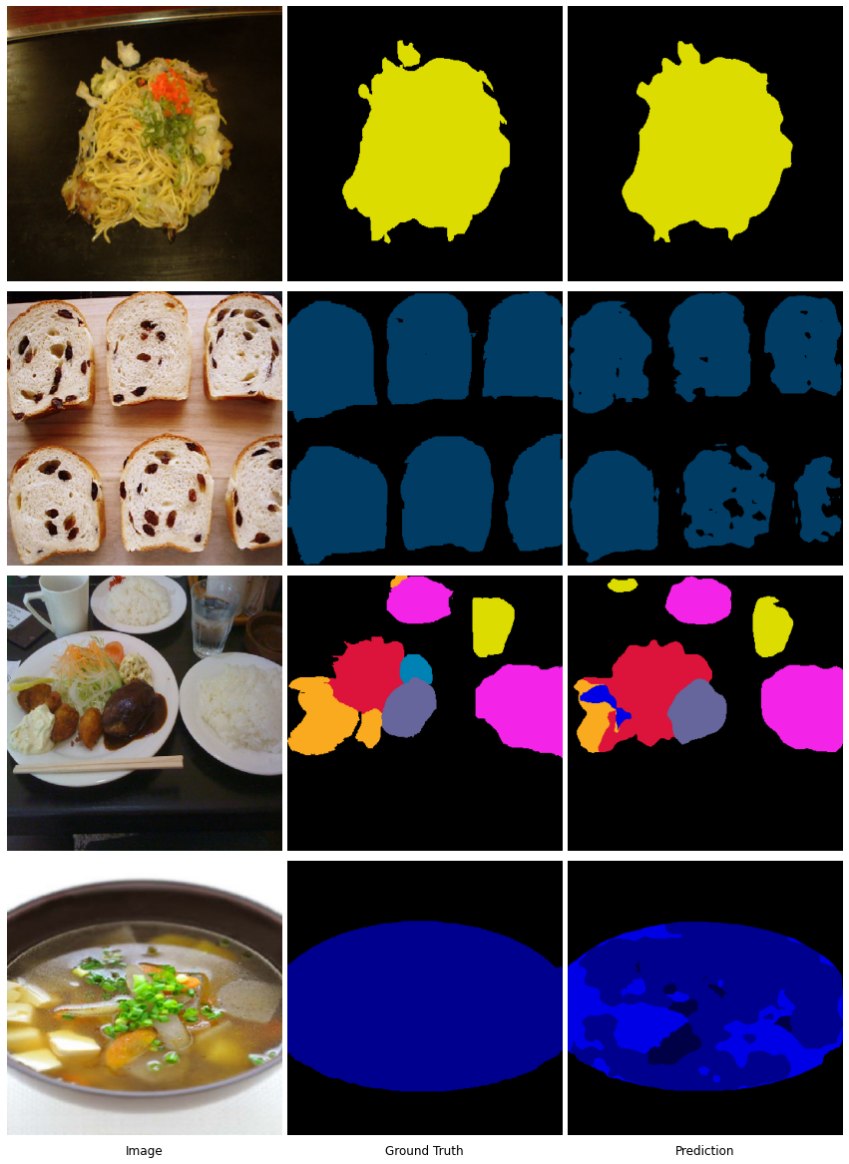
Sample images, ground truth masks and corresponding predictions from the UEC FoodPix dataset.

We next performed testing on the UEC FoodPix dataset, which is more challenging due to occurrences of multiple food items in proximity, different angles, and different resolutions for training and testing images. The mIOU results are shown in Table 4. GourmetNet outperforms the current state-of-the-art achieving 65.13% mIOU, a significant performance increase of 5.8% compared to DeepLabv3+ and 17.2% compared to the dataset baseline set by [27]. The examples in Figure 7 demonstrate successful segmentations for the UEC FoodPix dataset. These examples show that GourmetNet deals effectively with food accuracy, localization, and shape. Challenging conditions are due to different food types overlapping and in close proximity or with different items composing a single dish, for example, a bowl of soup containing vegetables and tofu in its broth.

### 5.3. Food Classes Performance Analysis

Table 5 lists the performance of GourmetNet for different food classes at both ends of the performance spectrum for the UEC FoodPix dataset. Food items that present constant shape and color, that are displayed with separation from other items, present a more solid consistency and achieve a higher mIOU from the GourmetNet model. Examples of classes containing these characteristics are croquette and pancakes. Another important factor for high accuracy is the fact that the class is visually distinct from the other classes, that is, udon noodle and goya chanpuru. Food classes that are routinely served in a separate bowl, such as mixed rice, also achieve a high mIOU score.

On the low performing side of Table 5, classes that present food items in close proximity to other food items have the lowest scores. For example, fried fish has a significant overlap and cross-error with other fried food items. A similar cross-error is observed for tempura and vegetable tempura, as well as chip butty being more routinely mistaken with other types of chips from the dataset. Another source of error is the presence of sauces or garnishing, altering the shape and color of the food item, and consequently increasing its variability. One example of this occurrence is salmon meunière.

## 6. Conclusions

We presented GourmetNet, a novel, end-to-end trainable architecture for food segmentation. GourmetNet incorporates the benefits of feature refinement from the channel and attention modules with the improved multi-scale feature representations of the WASPv2 module. The GourmetNet model expands semantic segmentation to the food domain and achieves state-of-the-art results on food segmentation datasets.

The goal of GourmetNet is to achieve improved food segmentation accuracy, consequently improving the performance of related tasks, such as automatic nutrition monitoring, food volume estimation, recipe extraction, or meal preparation. In future work, the GourmetNet framework can be improved by making the process more computationally efficient and increasing segmentation accuracy, so that food segmentation can be incorporated in a larger system for food volume estimation for dietary recommendations or assistance for meal preparation.

## Figures and Tables

**Table 1:** Results of GourmetNet ablation experiments for various configurations on the UNIMIB2016 dataset. The segmentation accuracy is indicated by the mIOU score, while the model complexity is described by the number of parameters and GFLOPS.

Dual Attention	Channel Attention	Spatial Attention	ASPP	WASP	WASPv2	GFLOPs	#Params	mIOU
						87.20	47.95 M	68.25%
✓						51.56	45.58 M	69.44%
✓			✓			54.60	59.41 M	69.73%
✓				✓		46.98	47.49 M	69.25%
✓					✓	48.81	47.00 M	70.29%
					✓	47.02	46.9 M	69.17%
	✓				✓	53.62	48.7 M	70.28%
		✓			✓	72.00	46.9 M	70.58%
	✓	✓			✓	78.60	48.8 M	**71.79%**
✓	✓	✓			✓	78.60	49 M	69.79%

**Table 2:** Results of GourmetNet ablation experiments for various configurations on the UEC FoodPix dataset. The segmentation accuracy is indicated by the mIOU score, while the model complexity is described by the number of parameters and GFLOPS.

Dual Attention	Channel Attention	Spatial Attention	ASPP	WASP	WASPv2	GFLOPs	#Params	mIOU
						51.33	47.95M	62.33%
✓						30.21	45.58M	62.48%
✓			✓			31.89	59.41M	62.49%
✓				✓		27.47	47.49M	61.95%
✓					✓	28.91	47M	63.14%
					✓	27.5	46.9M	63.54%
	✓				✓	31.4	48.7M	64.30%
		✓			✓	42.3	46.9M	64.29%
	✓	✓			✓	46.2	48.8M	**65.13%**
✓	✓	✓			✓	31.9	49M	63.92%

**Table 3:** GourmetNet results and comparison with SOTA methods for the UNIMIB2016 dataset.

Method	mIOU
DeepLab [12]	43.3%
SegNet [11]	44%
WASPnet [6]	67.50%
DeepLabv3+ [37]	68.87%
GourmetNet (Ours)	71.79%

**Table 4:** GourmetNet results and comparison with SOTA methods for the UEC FoodPix dataset.

Method	mIOU
UEC FoodPix [27]	55.55%
DeepLabv3+ [37]	61.54%
WASPnet [6]	62.09%
GourmetNet (Ours)	65.13%

**Table 5:** Comparison and analysis of food segmentation performance class-wise for the UEC FoodPix dataset. The left section mentions classes with the highest mIOU while the right section mentions the classes with the lowest mIOU.

Food Name	mIOU	Food Name	mIoU
Croquette	92.16%	Fried Fish	16.29%
Pancake	91.67%	Tempura	17.46%
Udon Noodle	88.67%	Vegetable Tempura	18.23%
Goya Chanpuru	88.61%	Salmon Meuniere	30.28%
Mixed Rice	87.54%	Chip Butty	31.03%

## Data Availability

Not Applicable.

## Abbreviations

The following abbreviations are used in this manuscript:

ASPP	Atrous Spatial Pyramid Pooling
CE	Cross-Entropy
COCO	Common Objects in Context
DANet	Dual Attention Network
CRF	Conditional Random Fields
ENet	Efficient Network
FCN	Fully Convoluted Networks
IOU	Intersection over Union
JSEG	J measure based Segmentation
mIOU	Mean Intersection over Union
NLP	Natural Language Processing
PSPnet	Pyramid Scene Parsing Network
RNN	Recurrent Neural Network
SAP	Spatial Average Pooling
SGD	Stochastic Gradient Descent
SMP	Spatial Max Pooling
SPP	Spatial Pyramid Pooling
WASP	Waterfall Atrous Spatial Pooling
